# Robotic hand-sewn vs. laparoscopic linear-stapled Roux-en-Y gastric bypass: a propensity score-matched analysis of primary and conversion cases

**DOI:** 10.3389/fsurg.2026.1760249

**Published:** 2026-05-13

**Authors:** Moaz Abulfaraj

**Affiliations:** 1 Department of Surgery, Faculty of Medicine, King Abdulaziz University, Jeddah, Saudi Arabia; 2 Department of Surgery, Al Salama Hospital, Jeddah, Saudi Arabia

**Keywords:** hand-sewn anastomosis, laparoscopic surgery, linear-stapled anastomosis, propensity score matching, robotic surgery, Roux-en-Y gastric bypass

## Abstract

**Background:**

Roux-en-Y gastric bypass (RYGB) is performed as a primary procedure or as a conversion from sleeve gastrectomy for complications such as gastroesophageal reflux disease (GERD) or weight regain. Robotic hand-sewn gastrojejunostomy may improve precision compared with laparoscopic linear stapling, but comparative evidence remains limited. This study compared robotic hand-sewn and laparoscopic linear-stapled RYGB using propensity score matching (PSM).

**Methods:**

This retrospective cohort study included 67 patients undergoing primary or conversion RYGB at a Saudi tertiary center (2016–2024). After PSM, 26 robotic cases (11 primary, 15 conversion) were matched with 26 laparoscopic cases based on demographic and clinical variables. Outcomes included operative time, length of stay (LOS), postoperative pain, narcotic use, complications, costs, body mass index (BMI) reduction, and comorbidity improvement. Statistical significance was set at *p* < 0.05.

**Results:**

Matched groups were comparable (mean age 46 ± 8 years; BMI 41 ± 6 kg/m²). Robotic procedures had longer operative times (178 ± 25 vs. 158 ± 22 min, *p* < 0.001) and higher costs (21,500 vs. 11,500 Saudi riyals, *p* < 0.001). LOS was slightly shorter in the robotic group (1.8 ± 0.5 vs. 2.1 ± 0.5 days, *p* = 0.19). Pain scores were lower (2.1 ± 0.8 vs. 3.4 ± 1.1, *p* = 0.002), and narcotic use was reduced (32% vs. 69%, *p* = 0.01). Complication rates and 12-month outcomes were comparable.

**Conclusion:**

Robotic and laparoscopic RYGB show comparable efficacy and safety. The robotic approach reduces pain and narcotic use, with a trend toward shorter hospital stay, but increases operative time and costs, supporting selective use in complex cases.

## 1 Introduction

Roux-en-Y gastric bypass (RYGB) is a cornerstone bariatric procedure, performed either as a primary intervention for obesity or as a conversion from sleeve gastrectomy (SG) to address complications such as gastroesophageal reflux disease (GERD) or weight regain (1, 2). Although SG accounts for >50% of global bariatric surgeries, up to 20% of patients eventually require conversion because of suboptimal outcomes or adverse events (3, 4). RYGB, endorsed by the American Society for Metabolic and Bariatric Surgery (ASMBS) and the International Federation for the Surgery of Obesity and Metabolic Disorders (IFSO), offers durable weight loss and superior GERD control in primary and conversion cases. According to the 2022 ASMBS/IFSO guidelines, surgical eligibility includes a BMI ≥35 kg/m² regardless of the presence or severity of comorbidities or a BMI ≥30 kg/m² with metabolic disorders (5, 6).

Primary RYGB demands precise anastomosis, whereas conversion surgery is further complicated by adhesions and distorted anatomy (7). Although laparoscopy remains the standard approach, robotic systems offer enhanced dexterity and three-dimensional visualization, which may improve technical precision—particularly in hand-sewn gastrojejunostomy compared with laparoscopic linear stapling (8). Previous studies indicate potential advantages of robotic RYGB in reducing complications during primary procedures (9); however, comparative evidence for robotic hand-sewn vs. laparoscopic linear-stapled RYGB, particularly in conversion settings, remains limited (10–13). In Saudi Arabia, where obesity exceeds 35% and diabetes affects approximately one-quarter of adults, optimizing bariatric strategies is a public health priority (13). Although local reports show comparable outcomes between robotic and laparoscopic bariatric surgery, they lack propensity score matching (PSM) and detailed cost analyses (14, 15).

This study used PSM to compare robotic hand-sewn and laparoscopic linear-stapled RYGB performed for both primary and conversion indications. The analysis evaluated operative time, length of stay (LOS), complications, costs, weight loss, and comorbidity resolution. We hypothesized that both approaches would demonstrate comparable efficacy, with the robotic technique offering safety advantages offset by increased operative time and resource utilization.

## 2 Materials and methods

### 2.1 Study design and population

This retrospective cohort study included 67 patients who underwent primary or conversion RYGB at King Abdulaziz University, Jeddah, Saudi Arabia, between 2016 and 2024. Patient selection followed the ASMBS and IFSO criteria: primary RYGB for individuals with a BMI ≥35 kg/m² regardless of the presence or severity of comorbidities or a BMI ≥30 kg/m² with metabolic disorders and conversion RYGB after sleeve gastrectomy (SG) for GERD, weight regain (>10% from nadir), or suboptimal weight loss (<20% at 18 months) (5). One patient underwent laparoscopic RYGB for a post-SG anastomotic leak, which was subsequently complicated by a re-leak from the gastrojejunostomy. This patient was excluded from the study due to an unusually prolonged hospital stay of 3 months, which would have substantially biased LOS comparisons. All patients underwent preoperative esophagogastroduodenoscopy (EGD), imaging, and psychological evaluation.

**Figure 1:**
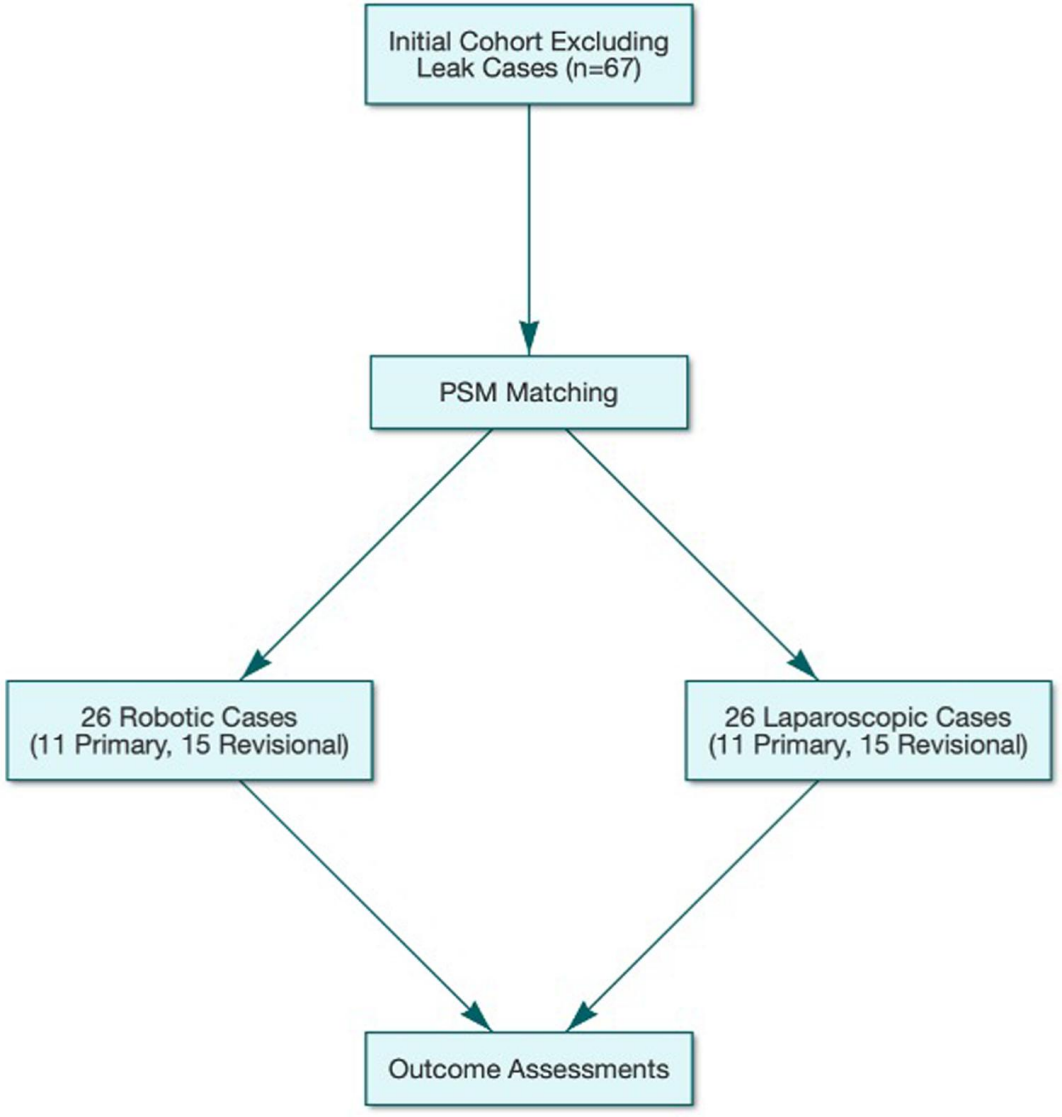
Flowchart of patient selection and propensity score matching process. The figure illustrates the initial cohort (*n* = 67), exclusion of cases with leaks, propensity score matching of 26 robotic (11 primary, 15 conversion) and 26 laparoscopic cases, and subsequent outcome assessments.

PSM was performed to match 26 robotic cases (11 primary and 15 conversion; 2022–2024, da Vinci Xi system) with 26 laparoscopic cases (2016–2021). Matching variables included age, sex, pre-RYGB BMI, comorbidities (diabetes, hypertension, and GERD), and procedure type (Figure 1). The nearest-neighbor algorithm with a caliper width of 0.2 was applied using R software (version 4.2.1).

### 2.2 Surgical technique

Laparoscopic RYGB was performed using five ports, including a liver retractor port, arranged in a semicircular upper abdominal configuration for optimal triangulation. Robotic RYGB employed four ports with a liver hammock stitch for retraction; a 12-mm assist port was added during the early learning phase. A 4–6 cm gastric pouch was created along the lesser curvature over a 40 Fr bougie, preserving the left gastric artery to maintain vascular supply. Hiatal hernias, when present, were repaired with nonabsorbable braided sutures, occasionally reinforced with mesh.

The jejunum was divided 75–100 cm distal to the ligament of Treitz to create the biliopancreatic limb, with limb length tailored to BMI and metabolic requirements—shorter in primary cases and longer in conversion cases, particularly for diabetes management (16). The Roux limb (75–100 cm) was brought antecolically. In the laparoscopic approach, the gastrojejunostomy was constructed using Endo GIA linear staplers (Medtronic) to form an approximately 2.5-cm anastomosis allowing smooth passage of the 40 Fr bougie. The common enterotomy was then closed with 3-0 barbed absorbable sutures for hemostasis; a hand-sewn laparoscopic anastomosis was not performed due to technical constraints. In the robotic approach, the gastrojejunostomy was hand-sewn in two layers using 3-0 barbed absorbable sutures to create a similar 2.5-cm anastomosis. The jejunojejunostomy was stapled in both groups, and all mesenteric defects were closed with running nonabsorbable sutures. Anastomotic integrity was confirmed intraoperatively using methylene blue testing. All procedures were performed by two surgeons with experience in over 500 bariatric operations and formal fellowship training in both robotic and bariatric surgery.

### 2.3 Data collection and outcomes

Data were obtained from electronic medical records and included demographics, preoperative and 12-month postoperative BMI, surgical indications, comorbidities, operative time, LOS, complications, and direct hospital costs [in Saudi riyals (SAR) and US dollar equivalents]. Short-term outcomes (≤30 days) comprised postoperative bleeding, port site hernia formation, and anastomotic stricture. At 12 months, evaluated outcomes included BMI reduction, GERD resolution—defined as symptom-free status without proton pump inhibitors and EGD confirmation (17)—diabetes remission (hemoglobin A1c < 6.5% without antidiabetic medication) (18), and hypertension improvement defined by reduced antihypertensive requirements (19).

### 2.4 Statistical analysis

Descriptive statistics were presented as means ± standard deviation for continuous variables and as frequencies and percentages for categorical variables. PSM balance was assessed using standardized mean differences, with values <0.1 indicating adequate matching. Paired *t*-tests were used to compare continuous outcomes, while chi-squared or Fisher's exact tests were applied for categorical variables. Analysis of variance (ANOVA) was used to evaluate differences between primary and conversion procedures. Statistical significance was defined as *p* < 0.05. All analyses were performed using SPSS software (version 26; IBM Corp., Armonk, NY, USA) and R (version 4.2.1).

## 3 Results

After PSM, 26 robotic cases (11 primary and 15 conversion) and 26 laparoscopic cases were well balanced, with standardized mean differences <0.1 across all variables. The mean patient age was 47 ± 8 years, 59% were female, and the preoperative BMI averaged 42 ± 6 kg/m². Among conversion cases, the primary indications were GERD (41%), weight regain (34%), and combined causes (25%), with a mean interval of 4.1 ± 2.0 years between SG and RYGB. Comorbidities included diabetes (49%), hypertension (36%), and GERD (61%) (Table 1).

**Table 1:** Baseline characteristics of patients after PSM.

Characteristic	Robotic (*n* = 26)	Laparoscopic (*n* = 26)	*p*-value
Age (years)	47 ± 8	46 ± 8	0.62
Female (%)	58%	58%	1.00
Pre-RYGB BMI (kg/m^2^)	42 ± 6	41 ± 6	0.95
Diabetes (%)	50%	46%	0.78
Hypertension (%)	31%	35%	0.76
GERD (%)	61%	61%	1.00
Procedure type: primary/conversion (%)	42/58	42/58	1.00
Conversion indications: GERD/weight regain/combined (%)	40/35/25	40/35/25	1.00

### 3.1 Short-term outcomes

Robotic hand-sewn procedures had significantly longer operative times (178 ± 25 vs. 158 ± 22 min, *p* < 0.001) and higher costs [21,500 ± 2,000 SAR [$5,733 ± 533 USD] vs. 11,500 ± 1,000 SAR [$3,067 ± 267 USD], *p* < 0.001]. Hospital LOS was slightly shorter in the robotic group (1.8 ± 0.5 vs. 2.1 ± 0.5 days, *p* = 0.19), while postoperative pain scores were lower [2.1 ± 0.8 vs. 3.4 ± 1.1 on the visual analog scale (VAS) at 24 h, *p* = 0.002] and narcotic requirements reduced (32% vs. 69% requiring opioids beyond POD1, *p* = 0.01). Complication rates were comparable between groups [7.7% in the robotic group [1 port site hernia, 1 stricture] vs. 11.5% in the laparoscopic group [2 bleeds, 1 stricture], *p* = 0.69]. No mortalities or intraoperative conversions occurred. Subgroup analysis revealed no significant differences between primary and conversion cases (Table 2).

**Table 2:** Comparison of short-term outcomes between robotic hand-sewn and laparoscopic linear-stapled RYGB after PSM.

Outcome	Robotic (hand-sewn)	Laparoscopic (linear-stapled)	*p*-value
Operative time (min)	178 ± 25	158 ± 22	<0.001
LOS (days)	1.8 ± 0.5	2.1 ± 0.5	0.19
Pain score (VAS at 24 h)	2.1 ± 0.8	3.4 ± 1.1	0.002
Narcotic use > POD1 (%)	32% (8/26)	69% (18/26)	0.01
Complications (%)	7.7% (2/26)	11.5% (3/26)	0.69
Cost (SAR [USD])	21,500 ($5,733) ± 2,000	11,500 ($3,067) ± 1,000	<0.001

### 3.2 12-month outcomes

BMI reduction (15 ± 3 vs. 14 ± 3 kg/m², *p* = 0.23), GERD resolution [89% [14/16] vs. 82% [13/16], *p* = 0.69], diabetes remission [92% [12/13] vs. 83% [10/12], *p* = 0.56], and hypertension improvement [75% [6/8] vs. 70% [7/10], *p* = 0.99] were comparable between groups. No significant differences were observed between primary and conversion cases (ANOVA, *p* > 0.05) (Table 3).

**Table 3:** Comparison of 12-month outcomes between robotic hand-sewn and laparoscopic linear-stapled RYGB after PSM.

Outcome	Robotic (hand-sewn)	Laparoscopic (linear-stapled)	*p*-value
BMI reduction (kg/m²)	15 ± 3	14 ± 3	0.23
GERD resolution (%)	89% (14/16)	82% (13/16)	0.69
Diabetes remission (%)	92% (12/13)	83% (10/12)	0.56
Hypertension improvement (%)	75% (6/8)	70% (7/10)	0.99

## 4 Discussion

This PSM study demonstrated comparable efficacy and safety for robotic hand-sewn and laparoscopic linear-stapled RYGB for both primary and conversion indications, excluding leak cases to avoid bias. Robotic surgery was associated with lower postoperative pain scores and reduced narcotic use, likely attributable to reduced abdominal wall trauma from static port placement and the absence of torque associated with conventional laparoscopic instruments (20). Both groups were subjected to the same postoperative analgesia protocol—scheduled intravenous acetaminophen and nonsteroidal anti-inflammatory drugs, with opioids reserved for breakthrough pain—and VAS scores were recorded every 12 h by nursing staff using a standardized numeric scale. No regional nerve blocks or adjunct nonopioid agents (e.g., ketamine, dexamethasone) were used in either cohort. Importantly, no significant changes were introduced to the postoperative recovery protocol between the two eras: both cohorts received the same multimodal analgesia regimen, early oral intake, and early ambulation targets. These uniform practices make it unlikely that perioperative care differences, rather than surgical technique itself, account for the observed reductions in pain and narcotic use, although residual confounding due to temporal separation cannot be excluded.

The robotic group had a marginally shorter length of hospital stay (1.8 vs. 2.1 days, *p* = 0.19), but the difference was not significant. However, operative time (178 vs. 158 min, *p* < 0.001) and costs (21,500 vs. 11,500 SAR, *p* < 0.001) were higher, reflecting the greater precision and resource demands of robotic hand-sewn anastomosis (8). Laparoscopic hand-sewn gastrojejunostomy was not attempted owing to limited instrument articulation, reinforcing the predominance of linear stapling in laparoscopic RYGB (21, 22).

Previous studies have reported conflicting findings regarding robotic vs. laparoscopic RYGB (Table 4). Ataya et al. observed that robotic surgery did not offer any advantages in complication rates or LOS among 5,809 conversion cases—mostly stapled procedures—with a higher risk of conversion (22). By contrast, Jawhar et al. emphasized the dexterity and precision of robotic systems in technically demanding cases, particularly those employing hand-sewn techniques (20). Spurzem et al. analyzed over 41,000 conversion cases from the MBSAQIP database and found that robotic conversion RYGB was associated with significantly lower overall morbidity, fewer blood transfusions, less surgical site infection, and shorter length of stay than the laparoscopic approach, despite longer operative times (23). Wise et al., in the largest single-center propensity-matched study to date, similarly showed less blood loss and shorter LOS in robotic primary bypass and sleeve, with no significant difference in adverse events or readmission (24). Tsenteradze et al. reported that robotic sleeve-to-RYGB conversion was associated with significantly shorter operative times and a lower late reoperation rate than laparoscopic conversion (25). Rapacz et al. evaluated >230,000 RYGB patients from the MBSAQIP database and reported no difference in 30-day complication rates between robotic and laparoscopic approaches, even in the high-risk BMI ≥60 kg/m² subgroup, with robotic procedures associated with a shorter length of stay despite longer operative times (26). Coco et al., in a 25-year meta-analysis of 38,647 patients from 27 countries, confirmed that robotic RYGB was associated with longer operative times but offered superior intraoperative safety including reduced blood loss and lower conversion rates, with comparable 30-day mortality, major complication rates, and long-term weight loss and metabolic outcomes (27). In the present study, the robotic hand-sewn anastomosis (∼2.5 cm, 3-0 barbed suture, 40 Fr bougie) demonstrated a low stricture rate (3.8%), although the laparoscopic linear-stapled approach produced comparable overall complication rates (28).

**Table 4:** Summary of key studies comparing robotic and laparoscopic bariatric surgery (primary and conversion).

Study (author, year)	Study type	Sample size (robotic/laparoscopic)	Key outcomes assessed	Main findings
Ataya et al., 2023 (22)	Systematic review and meta-analysis	5,809/55,889 (mixed conversion bariatric)	Leak, bleeding, operative time, LOS, surgical site infection (SSI), mortality, conversion, reoperation, readmission	No significant differences in leak (OR 0.94), bleeding (OR 2.48), operative time (MD −29.77 min), LOS (MD 0.37 days), SSI (OR 0.94), mortality (OR 2.82), or complications (OR 1.02); robotic approach had higher robotic conversion, reoperation, and readmission rates; most procedures were stapled
Jawhar et al., 2024 (20)	Narrative review	Varied (primary/conversion bariatric)	Operative time, morbidity, complications	Robotic surgery was safe with longer operative times and comparable morbidity; robotic hand-sewn anastomoses provided advantages in complex conversion cases due to enhanced dexterity
Spurzem et al., 2024 (23)	Retrospective database study (MBSAQIP)	41,404 conversion cases (SG + RYGB, 2015–2022)	Overall morbidity, blood transfusion, SSI, LOS, operative time	Robotic conversion RYGB exhibited significantly lower overall morbidity (4.8% vs. 6.2%), fewer blood transfusions, less SSI, and shorter LOS than the laparoscopic approach; operative time was longer with robotic; robot utilization increased from 7.3% to 32.0% over the study period
Wise et al., 2025 (24)	Retrospective single-center PSM study	5,690 patients (robotic vs. laparoscopic; primary and conversion, 2011–2023)	Blood loss, LOS, procedure time, readmission, adverse events	Robotic primary bypass and sleeve exhibited significantly less blood loss and shorter LOS; procedure time was significantly longer in all robotic groups; no significant difference in adverse events or readmission rates was observed.
Tsenteradze et al., 2025 (25)	Retrospective cohort study	126 sleeve-to-RYGB conversions (27 laparoscopic, 99 robotic)	Operative time, complications, rehospitalization, reintervention, weight loss, comorbidity resolution	Robotic conversion exhibited significantly shorter operative time (184 vs. 216 min, *p* < 0.001); late reoperation rate was significantly lower in robotic group (13.1% vs. 29.6%, *p* = 0.041); other early outcomes were comparable.
Rapacz et al., 2025 (26)	Retrospective database study (MBSAQIP)	233,828 RYGB patients (BMI <60 vs. ≥60 kg/m², 2020–2023)	30-day complications, ICU admission, renal failure, LOS, operative time	Patients with BMI ≥60 kg/m² exhibited higher complication rates; no difference in 30-day complication rate was observed between robotic and laparoscopic RYGB in the BMI ≥60 group; the robotic approach was associated with longer operative time but shorter LOS in this high-risk subgroup.
Coco et al., 2025 (27)	Systematic review and meta-analysis (25-year)	38,647 patients from 27 countries (42 comparative studies, 2000–2025)	Operative time, blood loss, conversion rate, 30-day mortality, major complications, 1-year EWL, 5-year diabetes remission	Robotic RYGB required longer operative time (+38.7 min), but exhibited less blood loss (−28.3 mL) and lower conversion rate (0.8% vs. 1.1%); 30-day mortality and major complication rates were comparable; 1-year EWL and 5-year diabetes remission were similar

Robotic operative times reflect the additional docking and hand-sewing steps but technically improve with experience (28). The shorter LOS observed in robotic cases likely indicates reduced tissue trauma and more stable port dynamics (20). Overall complication rates (7.7%–11.5%) fall within published norms (3%–15%) (7). Higher costs mainly stem from robotic equipment use and suturing duration, which may be offset in high-volume centers with optimized workflows (29). At 12 months, the favorable outcomes—BMI reduction (13–14 kg/m²) and high-resolution rates for GERD (89%–82%) and diabetes (80%–92%)—surpass regional benchmarks (14, 15), reflecting standardized surgical technique and comprehensive multidisciplinary care.

### 4.1 Strengths and limitations

This study's strengths include a PSM design, inclusion of primary and conversion RYGB cases, and a specific focus on anastomosis techniques. Moreover, it represents the first matched analysis of this type in the Middle East. However, this study also has a few limitations, including its retrospective nature, the position of robotic cases along the surgeon's learning curve, and the temporal separation of cohorts (robotic cases 2022–2024; laparoscopic cases 2016–2021). Although PSM adjusts for measured baseline covariates, it cannot completely account for secular trends such as evolving Enhanced Recovery After Surgery protocols, advances in anesthesia, and surgeon experience over time, which may independently influence outcomes such as pain scores, narcotic use, and LOS. The modest sample size (*n* = 52), which was determined by institutional case volume rather than a formal power calculation, provides approximately 80% power to detect a 20-min difference in operative time, but it may be insufficient to detect clinically meaningful differences in complication rates or 12-month outcomes. Thus, the comparable complication and efficacy results should be interpreted with caution. Additional limitations include the single-center setting and the absence of quality-of-life assessment. Thus, future multicenter randomized controlled trials with long-term follow-up are warranted to validate these findings (30).

## 5 Conclusion

Robotic hand-sewn and laparoscopic linear-stapled RYGB demonstrate comparable efficacy and safety in primary and conversion settings. Robotic surgery provides less postoperative pain and reduced narcotic use but has a longer operative time and higher expenses. Selective robotic use appears justified for complex cases requiring precise suturing, with larger prospective studies needed to confirm long-term clinical and economic benefits.

## Data Availability

The raw data supporting the conclusions of this article will be made available by the authors, without undue reservation.
